# Causal associations between chronic viral hepatitis and psychiatric disorders: a Mendelian randomization study

**DOI:** 10.3389/fpsyt.2024.1359080

**Published:** 2024-05-31

**Authors:** Haoshuang Fu, Shaowen Jiang, Shuying Song, Chenxi Zhang, Qing Xie

**Affiliations:** Department of Infectious Diseases, Ruijin Hospital, Shanghai Jiao Tong University School of Medicine, Shanghai, China

**Keywords:** Mendelian randomization, chronic hepatitis B, chronic hepatitis C, psychiatric disorders, schizophrenia, coffee consumption, underweight

## Abstract

**Background:**

There may be an interaction between viral hepatitis and psychiatric disorders during disease progression. Herein, we conducted Mendelian randomization (MR) to explore the causal associations and mediators between viral hepatitis and psychiatric disorders.

**Methods:**

Genome-wide association studies summary data for viral hepatitis [including chronic hepatitis B (CHB) and chronic hepatitis C (CHC)] and psychiatric disorders (including depression, anxiety, schizophrenia, obsessive-compulsive disorder, bipolar disorder, and post-traumatic stress disorder) were obtained. Two-sample MR was performed to assess the causal associations between viral hepatitis and psychiatric disorders. Further, a mediation analysis was conducted to evaluate the potential mediators. Inverse-variance weighted, MR-Egger, and weighted median were used as the main methods, while a sensitivity analysis was performed to evaluate pleiotropy and heterogeneity.

**Results:**

There was no causal effect of CHB/CHC on psychiatric disorders, as well as psychiatric disorders on CHB. However, schizophrenia presented a causal effect on increased CHC risk [odds ratio (OR)=1.378, 95%CI: 1.012–1.876]. Further, a mediation analysis identified coffee consumption and body mass index as mediators in the effect of schizophrenia on CHC, mediating 3.75% (95%CI: 0.76%–7.04%) and 0.94% (95%CI: 0.00%–1.70%) proportion, respectively.

**Conclusion:**

We revealed that schizophrenia patients faced a high risk of CHC, and insufficient coffee consumption and underweight could mediate the causal effect of schizophrenia on CHC. The prevention of hepatitis C might be a beneficial strategy for patients with schizophrenia. The right amount of nutrition supplements and coffee consumption might be part of a beneficial lifestyle in preventing the high CHC risk in patients with schizophrenia.

## Introduction

Viral hepatitis, particularly hepatitis B and C, are the leading etiologies of cirrhosis and liver cancer globally (1). They constitute a significant threat for human health, leading to 25% of deaths among patients with infectious diseases worldwide (1).

Hepatitis B virus is transmitted through contact with infected blood or semen (2). Hepatitis C virus is transmitted through common routes including unsafe skin-penetrating health-care practices, transfusion of unscreened blood and blood products, and injection drug use (3). Less common routes of transmission include vertical and sexual transmission (3). Multiple factors contribute to the disparity of hepatitis viral infection outcomes, including host factors, viral factors and environmental factors. A number of determinants in the chromosomes, including mutations in human leukocyte antigens, cytokines genes, toll-like receptors, and other genes are related to the human susceptibility to hepatitis B virus infection (4). Moreover, it was reported that human leukocyte antigens and single nucleotide polymorphisms in the IFNL3 gene locus were associated with both spontaneous clearance and clearance via interferon-based antiviral treatment (5). It was reported that tobacco, alcohol use, and a high body mass index were associated with the increasing burden of hepatitis B (6). Age, sex, high body mass index, and routes of infection were also associated with increasing burden of hepatitis C (7). However, achieving the elimination goal of the United Nations by the year 2023 seems unattainable (8). Therefore, it is imperative to explore the risk factors and progression for viral hepatitis.

Psychiatric disorders have been reported in the development and progression of viral hepatitis (9–16). It has been reported that patients with psychiatric disorders were at a high risk of viral hepatitis infections (9). Another study has found that patients with serious mental illness showed high hepatitis C virus seroprevalence (10). Additionally, an increased risk of concurrent hepatitis C among male patients with schizophrenia has been observed (11). However, a recent study found no association between psychiatric disorders and viral hepatitis (12). Moreover, two studies found that patients with viral hepatitis faced the stigma, affecting their mental health and quality of life (13, 14). Besides, a recent study found that up to 50% of the patients with hepatitis C may experience cognitive decline and psychiatric disorders, such as depression and fatigue (15). Another study indicated a high prevalence of personality disorders in prisoners with hepatitis C (16). Given the limitations of the previous studies, including small population size, controversial interpretation, and confounding factors, such as obesity and age, there is an urgent need to determine the causal connections between viral hepatitis and psychiatric disorders.

The rapid development of genome-wide association studies (GWAS) has led to the increasing application of mendelian randomization (MR) analysis, which is an instrument variable (IV) method utilizing single nucleotide polymorphisms (SNPs) as instruments, has been employed to elucidate causal relationships between two features. MR is based on three hypotheses: (1) the selected instrument variable is strongly associated with the exposure; (2) the selected instrument variable is not associated with confounders; and (3) the selected variable affects the outcome only through the exposure rather than other pathways. Since SNP is randomly assigned at conception, the results of MR offer two advantages: mitigating a bias resulting from the confounding factors and addressing the issues related to reverse causality (17).

Therefore, we performed a univariable MR (UVMR) analysis to explore the causal associations between viral hepatitis [including chronic hepatitis B (CHB) and chronic hepatitis C (CHC)] and psychiatric disorders [including depression, anxiety, schizophrenia, obsessive-compulsive disorder, bipolar disorder, and post-traumatic stress disorder (PTSD)]. Further, multivariable MR (MVMR) and mediation analysis were conducted to screen mediators and assess the mediation proportion.

## Methods

### Study design

**Figure 1:**
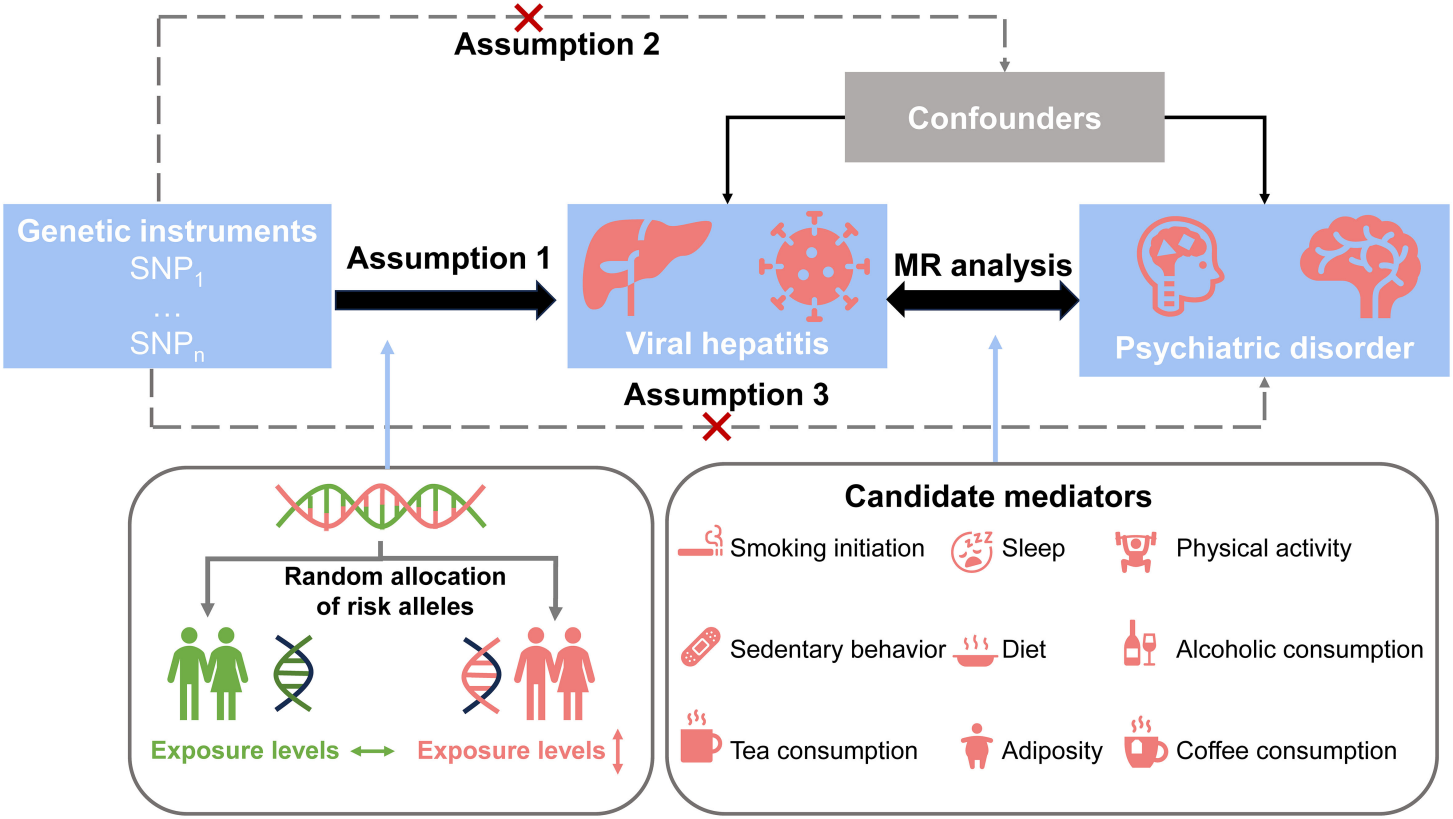
Study design. Study design overview.

The study design is shown in **Figure 1**. Initially, we identified SNPs as IVs and performed UVMR to explore the causal effect of chronic viral hepatitis on psychological disorders. Subsequently, we performed the analysis in reverse to assess the inverse causalities. Furthermore, we screened the mediators in the association between psychiatric disorders and chronic viral hepatitis from the lifestyle factors based on the mediator selection process, using UVMR and MVMR, and assessed the mediation proportion of screened mediators. Our study was in accordance with the Strengthening the Reporting of Observational Studies in Epidemiology reporting guidelines (18) (**Supplementary Materials**).

### Data sources

The GWAS data based on large-scale studies were used in this study (**Table 1**). Overlapping individuals have negligible effects on the power when IVs are strong enough (19). All procedures followed were in accordance with the ethical standards of the responsible committee on human experimentation (institutional and national) and with the Helsinki Declaration of 1975, as revised in 2008. All the studies included in the cited genome-wide association studies were approved by a relevant review board. All participants provided informed consent.

**Table 1:** Data sources.

Phenotype	Unit	Participants	Ancestry	Consortium/cohort	PMID/Web
Viral hepatitis					
Chronic hepatitis B	Odds ratio	145 cases and 351,740 controls	European	Meta	34594039
Chronic hepatitis C	Odds ratio	273 cases and 351,740 controls	European	Meta	34594039
Psychiatric disorders					
Depression	Odds ratio	170,756 cases and 329,443 controls	European	Meta	30718901
Anxiety	Odds ratio	5580 cases and 11,730 controls	European	Meta	26754954
Schizophrenia	Odds ratio	53,386 cases and 77,258 controls	European	Psychiatric Genomics Consortium	35396580
Obsessive-compulsive disorder	Odds ratio	2688 cases and 7037 controls	European	Meta	28761083
Bipolar disorder	Odds ratio	41,917 cases and 371,549 controls	European	Meta	34002096
Post-traumatic stress disorder	Odds ratio	23,212 cases and 151,447 controls	European	Meta	(https://doi.org/10.1101/458562)

All data were sourced from European ancestry. The data for CHB (145 cases and 351,740 controls) and CHC (273 cases and 351,740 controls) were obtained from a meta-analysis (20). The data for depression (21) (170,756 cases and 329,443 controls) and anxiety (22) (5580 cases and 11,7430 controls) were obtained from the meta-analyses, respectively. The data for schizophrenia, including 53,386 cases and 77,258 controls, were sourced from the Psychiatric Genomics Consortium (23). The data for obsessive-compulsive disorder (24), bipolar disorder (25) and PTSD (https://doi.org/10.1101/458562) were sourced from meta-analyses, including 2688 cases and 7037 controls, 41,917 cases and 371,549 controls, and 23,212 cases and 151,447 controls, respectively. The detailed information for diagnosis criteria and quality control is summarized in **Supplementary Table S1**.

We selected 25 lifestyle factors as candidate mediators, which could affect the associations between chronic viral hepatitis and psychiatric disorders and could be modifiable by interventions. The data for mediators were all sourced from European ancestry, including smoking, sleep, physical activity, sedentary behavior, diet, alcohol assumption, tea assumption, coffee consumption, and adiposity. The detailed information for GWAS-data of mediators is summarized in **Supplementary Table S2**.

### Selection of IVs

The SNPs for chronic viral hepatitis and psychiatric disorders were selected as IVs with a P-value (<5×10^-8^), whereas a relaxed threshold of P-value (<5×10^-6^) was used for CHB, CHC, anxiety, obsessive-compulsive disorder, and PTSD to obtain enough IVs. All SNPs were clumped for independent inheritance (R^2^<0.001, within 10 Mb). F-statistics were calculated to assess the validity of the SNPs, with a threshold exceeding 10. The LDtrait Tool (https://ldlink.nih.gov/?tab=ldtrait) was used to exclude the potential confounding factors (P<5×10^−8^). However, no confounding factor was detected.

### Screening of mediators

Based on the causal effects of schizophrenia on CHC, we screened the mediators from factors covering all aspects of life to provide beneficial life styles in preventing the high CHC risk in patients with schizophrenia (26), following the mediator selection criteria (26). Criterion 1: The mediator should have a causal effect on CHC in UVMR; Criterion 2: The mediator should have a direct causal effect on CHC controlled for schizophrenia in MVMR; Criterion 3: Schizophrenia should be causally associated with the mediator, but not vice versa; and Criterion 4: The associations of schizophrenia with the mediator and mediator with CHC should be in the same direction.

### Statistical analysis

Three methods [random-effect inverse-variance weighted (IVW), MR-Egger, and weighted median] were performed to estimate the causal associations between the exposures and outcomes, for which the assumptions and advantages are summarized in **Supplementary Table S3**. The primary method was IVW. In the presence of pleiotropy (P < 0.05), MR-Egger was the primary method (27). With over 50% valid genetic instruments, we used the weighted median for robust causal estimates (28). In UVMR and MVMR, Cochran’s Q statistic and MR-Egger intercept were performed to evaluate heterogeneity and pleiotropy, respectively. The MR-PRESSO was used to remove the outliers for horizontal pleiotropy and evaluate whether the exclusion of the outlying SNPs influences the causal estimates (27). In addition, an MRlap analysis was performed using the same IVs selection criteria, aiming to avoid biases introduced by a sample overlap, winner’s curse, and weak instruments (29). Additionally, the mediation proportion of the mediator was assessed using the product of coefficients method. All tests were two-sided and performed using the TwoSampleMR (version 0.5.7), MVMR (version 0.4), MRlap (version 0.0.3.0), and Mendelian Randomization (version 0.9.0) packages in the R software (version 4.0.2). A Bonferroni-corrected P-value less than 0.05/5 (0.01) was identified as statistically significant, and a P-value less than 0.05 was identified as nominally significant.

## Results

### Identification of IVs

The SNPs for CHB, CHC, and psychiatric disorders are summarized in **Supplementary Tables S4-S11**. Given that the F-statistics for obsessive-compulsive disorder was less than 10, it was only used as an outcome rather than exposure. All other IVs exceeded 10, indicating the sufficient validity of the SNPs. Next, the LDtrait Tool (https://ldlink.nih.gov/?tab=ldtrait) was used to exclude the potential confounding factors (P<5×10^−8^). However, no confounding factor was detected.

### Causal effects of chronic viral hepatitis on psychiatric disorders

**Figure 2:**
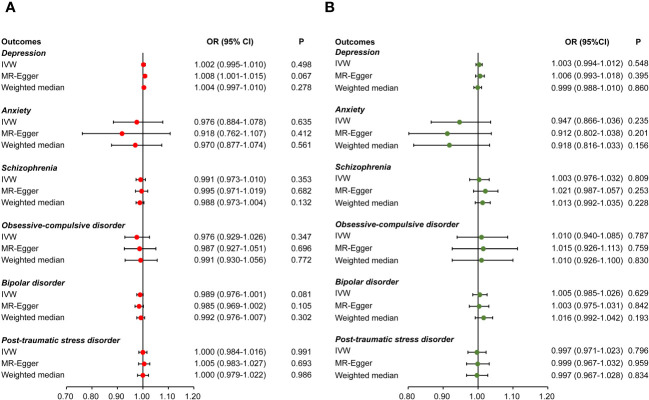
Causal effects of viral hepatitis on psychiatric disorders. Causal effects of chronic hepatitis B **(A)** and chronic hepatitis C **(B)** on psychiatric disorders, including depression, anxiety, schizophrenia, obsessive-compulsive disorder, bipolar disorder, and post-traumatic stress disorder.

The causal effects of CHB/CHC on psychiatric disorders are summarized in **Figure 2**. No causal effects of CHB/CHC on psychiatric disorders were observed using the IVW method, which were validated using the MRlap method (**Supplementary Table S12**), indicating that a sample overlap may not substantially affect the causal results. Heterogeneity was only detected in the effects of CHB on depression and schizophrenia, effects of CHC on schizophrenia, respectively (**Table 2**). Pleiotropy was only detected in effect of CHB on depression, whereas the effect persisted after correction using MR-PRESSO (**Table 2**). Outliers were only detected in the effects of CHB and CHC on schizophrenia, whereas the effects persisted after correction using MR-PRESSO.

**Table 2:** Sensitivity analysis for effects of viral hepatitis on psychiatric disorders.

Exposure	Outcome	Cochran’s Q	P_Cochran’s Q	P_intercept	MR-PRESSO analysis*				
					OR	95%CI	P	Outlier	P_global_test
Chronic hepatitis B	Depression	17.522	0.041	0.039	1.002	(0.995, 1.010)	0.515	0	0.100
	Anxiety	10.368	0.110	0.477	0.976	(0.884, 1.078)	0.651	0	0.231
	Schizophrenia	34.416	0.001	0.628	0.995	(0.979, 1.011)	0.552	1	0.002
	Obsessive-compulsive disorder	3.154	0.924	0.594	0.976	(0.946, 1.007)	0.172	0	0.925
	Bipolar disorder	13.619	0.326	0.513	0.989	(0.976, 1.001)	0.107	0	0.426
	Post-traumatic stress disorder	2.227	1.000	0.549	1.000	(0.993, 1.006)	0.977	0	1.000
Chronic hepatitis C	Depression	18.437	0.103	0.515	1.003	(0.994, 1.012)	0.559	0	0.112
	Anxiety	8.316	0.503	0.453	0.947	(0.869, 1.032)	0.248	0	0.626
	Schizophrenia	44.276	2.76E-05	0.141	1.006	(0.982, 1.030)	0.654	1	1.00E-04
	Obsessive-compulsive disorder	1.262	0.999	0.870	1.010	(0.983, 1.037)	0.488	0	0.994
	Bipolar disorder	21.046	0.072	0.810	1.005	(0.985, 1.026)	0.637	0	0.067
	Post-traumatic stress disorder	1.308	1.000	0.809	0.997	(0.989, 1.004)	0.379	0	1.000

*The MR-PRESSO estimates was outlier-corrected when the outlier was present.

### Causal effects of psychiatric disorders on chronic viral hepatitis

**Figure 3:**
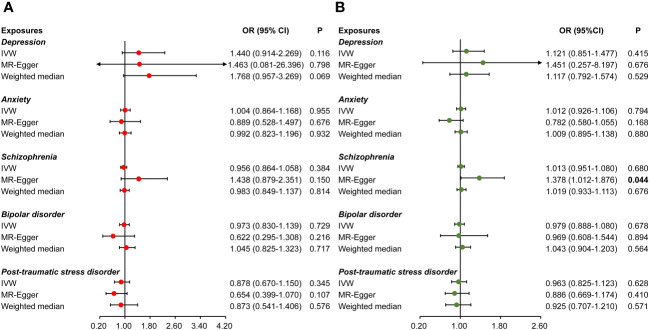
Causal effects of psychiatric disorders on viral hepatitis. Causal effects of psychiatric disorders (including depression, anxiety, schizophrenia, bipolar disorder, and post-traumatic stress disorder) on chronic hepatitis B **(A)** and chronic hepatitis C **(B)**.

The causal effects of psychiatric disorders on CHB/CHC are summarized in **Figure 3**. There was no causal effect of psychiatric disorders on CHB/CHC using the IVW method, which were validated using the MRlap method (**Supplementary Table S12**), indicating that a sample overlap may not substantially affect the causal results. However, given the existence of pleiotropy in the association between schizophrenia and CHC (**Table 3**), MR-Egger was used as the main method to assess their association. Notably, we found a nominal effect of schizophrenia on the increased risk of CHC [Odds ratio (OR) =1.378, 95%CI: 1.012–1.876, P=0.044, **Figure 3B**]. No pleiotropy was found in the other estimates (**Table 3**). Heterogeneity was only detected in the effect of depression on CHC (**Table 3**). No outlier was detected using MR-PRESSO (**Table 3**).

**Table 3:** Sensitivity analysis for effects of psychiatric disorders on viral hepatitis.

Exposure	Outcome	Cochran’s Q	P_Cochran’s Q	P_intercept	MR-PRESSO analysis*				
					OR	95%CI	P	Outlier	P_global_test
Depression	Chronic hepatitis B	60.233	0.078	0.991	1.440	(0.914, 2.269)	0.123	0	0.075
Anxiety		1.698	0.945	0.652	1.004	(0.927, 1.088)	0.919	0	0.949
Schizophrenia		145.951	0.509	0.099	0.956	(0.864, 1.058)	0.383	0	0.515
Bipolar disorder		35.177	0.964	0.233	0.973	(0.854, 1.107)	0.676	0	0.967
Post-traumatic stress disorder		17.508	0.680	0.176	0.878	(0.686, 1.124)	0.312	0	0.715
Depression	Chronic hepatitis C	62.304	0.045	0.769	1.121	(0.851, 1.477)	0.419	0	0.049
Anxiety		6.309	0.389	0.138	1.012	(0.926, 1.106)	0.802	0	0.390
Schizophrenia		173.644	0.066	0.049	1.013	(0.951, 1.080)	0.680	0	0.066
Bipolar disorder		60.487	0.196	0.962	0.979	(0.888, 1.080)	0.680	0	0.195
Post-traumatic stress disorder		18.753	0.601	0.499	0.963	(0.832, 1.113)	0.613	0	0.632

*The MR-PRESSO estimate was outlier-corrected when the outlier was present.

### Mediation analysis

**Figure 4:**
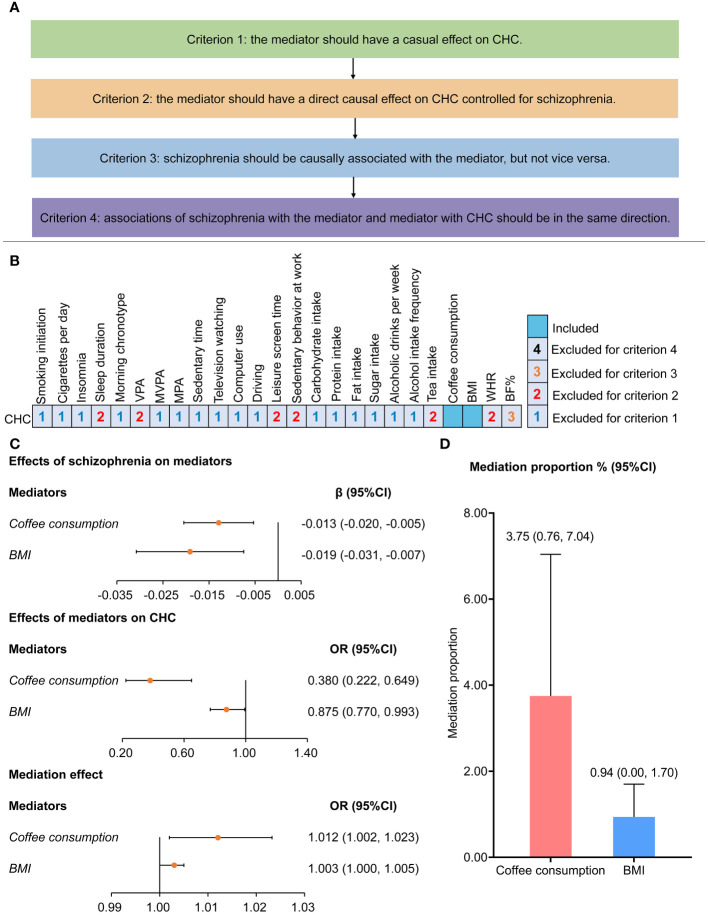
Screening process and assessment for mediators of effects of schizophrenia on chronic hepatitis B **(A)** Four criteria were applied to select the mediators in the effect of schizophrenia on chronic hepatitis C: Criterion 1: the mediator should have a causal effect on CHC in UVMR; Criterion 2: the mediator should have a direct causal effect on CHC controlled for schizophrenia in MVMR; Criterion 3: schizophrenia should be causally associated with the mediator, but not vice versa; and Criterion 4: associations of schizophrenia with the mediator and mediator with CHC should be in the same direction. **(B)** Among 25 lifestyle factors, coffee consumption and BMI were identified as mediators in association between schizophrenia and chronic hepatitis B **(C, D)** Two-step Mendelian randomization was performed to evaluate the mediation effect and proportion of coffee consumption and BMI in the causal association between schizophrenia and chronic hepatitis C BF%, body fat percentage; BMI, body mass index; CHC, chronic hepatitis C; MPA, moderate physical activity; MVMR, multivariable Mendelian randomization; MVPA, moderate to vigorous physical activity; UVMR, univariable Mendelian randomization; VPA, vigorous physical activity; WHR, waist-hip ratio.

We screened mediators in the association between schizophrenia and CHC from the life style factors based on the mediator selection process (**Figure 4A**). Most of the candidate mediators were excluded, whereas coffee consumption and body mass index (BMI) were identified as the mediators in the effect of schizophrenia on CHC (**Figure 4B**). Coffee consumption and BMI presented independent effects (OR=0.380, 95%CI: 0.222–0.649, **Figure 4C**) (OR=0.875, 95%CI: 0.770–0.993, **Figure 4C**) on decreased CHC risk after controlling for schizophrenia. Moreover, schizophrenia presented a causal effect on decreasing the coffee consumption (β=-0.013, 95%CI: -0.020 to -0.005, **Figure 4C**) and decreasing BMI (β=-0.019, 95%CI: -0.031 to -0.007, **Figure 4C**). A further mediation analysis showed that coffee consumption and BMI mediated 3.75% (95%CI: 0.76%–7.04%) and 0.94% (95%CI: 0.00%–1.70%) proportion in the effect of schizophrenia on increased risk of CHC, respectively (**Figure 4D**).

## Discussion

Our study systemically evaluated the causal associations between viral hepatitis and psychiatric disorders. We revealed the causal effect of schizophrenia on high CHC risk, whereas no causal association was observed between CHB and psychiatric disorders. A further mediation analysis found that coffee consumption and underweight were the mediators in the effect of schizophrenia on CHC risk.

No causal effects of CHB/CHC on psychiatric disorders and effects of psychiatric disorders on CHC were observed in our study. Several studies found that patients with viral hepatitis faced stigma, affecting their self-esteem and quality of life, even resulting in depression (13, 14, 30, 31). However, we found no evidence for the influence of viral hepatitis on the mental health of patients. It has been found that depression in patients with CHB was related to the use of antiviral drugs and interferon alpha, rather than the hepatitis B virus infection (32, 33). Moreover, a similar mechanism has been reported in patients with CHC with the combined therapeutics using Pegylated IFN (34). which could result in the difference between our results and previous results.

Schizophrenia was the risk factor for CHC risk. Our results supported previous studies: patients with schizophrenia faced a high risk for CHC infection (9). An increasing susceptibility and prevalence of environmental risk factors could result in the effect of schizophrenia on CHC risk. Patients with schizophrenia were reported as the feature of abnormal immune cell function with a decreased natural killer cell activity (35), which could result in increasing susceptibility to infection. Besides, substance use comorbidity and risky sexual behavior were also reported as the underlying mechanisms of high CHC risk of patients with schizophrenia (35).

Our study identified coffee consumption and BMI as the novel modified targets for the prevention of CHC in patients with schizophrenia. Among 25 lifestyle factors, coffee consumption and BMI were screened as the mediators in the effect of schizophrenia on CHC risk. It has been reported that coffee consumption could increase the telomere length and decrease the oxidative DNA damage in patients with CHC, reducing the progression of disease (36). We concluded the same result: coffee consumption could reduce the CHC risk, while patients with schizophrenia faced decreasing coffee consumption, resulting in an increase of the CHC risk. Moreover, underweight was also identified as a potential mediator in the causal effect of schizophrenia on CHC risk. Underweight indicates potential malnutrition, which could affect the development and function of the immune system (37), resulting in the infection of various pathogens. Notably, we found that schizophrenia patients faced decreasing BMI and increasing malnutrition, resulting in increasing of the CHC risk. Although their mediation proportions were low, our results showed that the proportions mediated were statistically significant. Besides, a previous study also indicated the low mediation proportions of several mediators (26). Although the right amount of nutrition supplements and coffee consumption might not be able to make a milestone change in preventing the high CHC risk in patients with schizophrenia, it might provide a more beneficial lifestyle for patients with schizophrenia.

There are several strengths in our study. First, we conducted an MR analysis using large-scale GWAS data, which could mitigate the bias resulting from confounding factors and address the issues related to reverse causality (17). Second, we identified that the right amount of nutrition supplement and coffee consumption could be the novel targets for the prevention of CHC in patients with schizophrenia. Third, we limited our study to European ancestry to avoid bias in the population structure.

This study has a few limitations, which should be considered when interpreting our results. First, the selection for partial exposures was conducted on a relax threshold (<5×10^-6^). However, it was reassuring that the IVs’ F-statistics exceeded 10, indicating that the weak instrument bias should be minimal. Second, partial nominal results in our study were limited to the MR-Egger and weighted median methods rather than the IVW method. Thus, our results should be interpreted cautiously, and further study is required to validate our findings. Third, the study population of our study were limited to European ancestry, indicating that our results should be cautiously interpreted across non-European populations.

## Conclusion

The findings of this study revealed that patients with schizophrenia faced a high risk of CHC, and insufficient coffee consumption and underweight could mediate the causal effect of schizophrenia on CHC. The prevention of hepatitis C might be a beneficial strategy for patients with schizophrenia. The right amount of nutrition supplement and coffee consumption might be beneficial lifestyles in preventing high CHC risk in patients with schizophrenia.

## Data availability statement

The original contributions presented in the study are included in the article/**Supplementary Material**. Further inquiries can be directed to the corresponding authors.

## Ethics statement

All procedures followed were in accordance with the ethical standards of the responsible committee on human experimentation (institutional and national) and with the Helsinki Declaration of 1975, as revised in 2008. All studies included in the cited genome-wide association studies had approved by a relevant review board. All participants provided informed consent.

## Author contributions

HF: Conceptualization, Formal analysis, Investigation, Methodology, Writing – original draft. SJ: Methodology, Writing – review & editing. SS: Writing – review & editing, Formal analysis, Investigation. CZ: Conceptualization, Writing – review & editing. QX: Conceptualization, Writing – review & editing.
