# Supplementary material for: Causal associations between chronic viral hepatitis and psychiatric disorders: a Mendelian randomization study
Source: Front Psychiatry. 2024 May 31;15:1359080. doi: 10.3389/fpsyt.2024.1359080 (PMC11176532; doi:10.3389/fpsyt.2024.1359080)
Supplement: Supplementary file 1 [file Table_1.docx]

**STROBE-MR checklist of recommended items to address in reports of Mendelian randomization studies**^1^ ^2^

| **Item No.** | **Section** | **Checklist item** | **Page No.** | **Relevant text from manuscript** |
| --- | --- | --- | --- | --- |
| 1 | **TITLE and ABSTRACT** | Indicate Mendelian randomization (MR) as the study’s design in the title and/or the abstract if that is a main purpose of the study | - | Causal associations between chronic viral hepatitis and psychiatric disorders: A Mendelian randomization study |
|  | **INTRODUCTION** |  |  |  |
| 2 | **Background** | Explain the scientific background and rationale for the reported study. What is the exposure? Is a potential causal relationship between exposure and outcome plausible? Justify why MR is a helpful method to address the study question |  | Introduction |
| 3 | **Objectives** | State specific objectives clearly, including pre-specified causal hypotheses (if any). State that MR is a method that, under specific assumptions, intends to estimate causal effects | - | Therefore, we performed univariable Mendelian randomization (UVMR) to explore the causal associations between viral hepatitis [including chronic hepatitis B (CHB) and chronic hepatitis C (CHC)] and psychological disorders [including depression, anxiety, schizophrenia, obsessive-compulsive disorder, bipolar disorder, and post-traumatic stress disorder (PTSD)], further multivariable Mendelian randomization (MVMR) and mediation analysis were conducted to screen mediators and assess the mediation proportion.. |
|  | **METHODS** |  |  |  |
| 4 | **Study design and data sources** | Present key elements of the study design early in the article. Consider including a table listing sources of data for all phases of the study. For each data source contributing to the analysis, describe the following: |  |  |
|  | a) | Setting: Describe the study design and the underlying population, if possible. Describe the setting, locations, and relevant dates, including periods of recruitment, exposure, follow-up, and data collection, when available. | - | Figure 1 and Table 1 |
|  | b) | Participants: Give the eligibility criteria, and the sources and methods of selection of participants. Report the sample size, and whether any power or sample size calculations were carried out prior to the main analysis | - | Table 1 |
|  | c) | Describe measurement, quality control and selection of genetic variants | - | Table 1 |
|  | d) | For each exposure, outcome, and other relevant variables, describe methods of assessment and diagnostic criteria for diseases | - | Table 1 |
|  | e) | Provide details of ethics committee approval and participant informed consent, if relevant | - | All procedures followed were in accordance with the ethical standards of the responsible committee on human experimentation (institutional and national) and with the Helsinki Declaration of 1975, as revised in 2008. All studies included in cited genome-wide association studies had approved by a relevant review board. All participants had provided the inform consent. |
| 5 | **Assumptions** | Explicitly state the three core IV assumptions for the main analysis (relevance, independence and exclusion restriction) as well assumptions for any additional or sensitivity analysis | - | MR is based on three hypotheses: (1) the selected instrument variable is strongly associated with the exposure; (2) the selected instrument variable is not associated with confounders; and (3) the selected variable affects the outcome only through the exposure rather than other pathways. Since SNP is randomly assigned at conception, the results of MR offers two advantages: mitigating a bias resulting from the confounding factors and addressing the issues related to reverse causality[17]. |
| 6 | **Statistical methods: main analysis** | Describe statistical methods and statistics used |  |  |
|  | a) | Describe how quantitative variables were handled in the analyses (i.e., scale, units, model) | - | Table 1 |
|  | b) | Describe how genetic variants were handled in the analyses and, if applicable, how their weights were selected | - | The SNPs for chronic viral hepatitis and psychiatric disorders were selected as IVs with a P-value (<5×10-8), whereas a relaxed threshold of P-value (<5×10-6) was used for CHB, CHC, anxiety, obsessive-compulsive disorder, and PTSD to obtain enough IVs. All SNPs were clumped for independent inheritance (R2<0.001, within 10 Mb). F-statistics were calculated to assess the validity of the SNPs, with a threshold exceeding 10. The LDtrait Tool (https://ldlink.nih.gov/?tab=ldtrait) was used to exclude the potential confounding factors (P<5×10−8). However, no confounding factor was detected. |
|  | c) | Describe the MR estimator (e.g. two-stage least squares, Wald ratio) and related statistics. Detail the included covariates and, in case of two-sample MR, whether the same covariate set was used for adjustment in the two samples | - | Table 1 |
|  | d) | Explain how missing data were addressed | - | - |
|  | e) | If applicable, indicate how multiple testing was addressed | - | - |
| 7 | **Assessment of assumptions** | Describe any methods or prior knowledge used to assess the assumptions or justify their validity | - | F-statistics were calculated to asses SNP’s validity, with a threshold of exceeding 10.. |
| 8 | **Sensitivity analyses and additional analyses** | Describe any sensitivity analyses or additional analyses performed (e.g. comparison of effect estimates from different approaches, independent replication, bias analytic techniques, validation of instruments, simulations) | - | Three methods [random-effect inverse-variance weighted (IVW), MR-Egger, and weighted median] were performed to estimate the causal associations between the exposures and outcomes, for which the assumptions and advantages are summarized in Table S3. The primary method was IVW. In the presence of pleiotropy (P < 0.05), MR-Egger was the primary method[27]. With over 50% valid genetic instruments, we used the weighted median for robust causal estimates[28]. In UVMR and MVMR, Cochran's Q statistic and MR-Egger intercept were performed to evaluate heterogeneity and pleiotropy, respectively. The MR-PRESSO was used to remove the outliers for horizontal pleiotropy and evaluate whether the exclusion of the outlying SNPs influences the causal estimates[27]. In addition, an MRlap analysis was performed using the same IVs selection criteria, aiming to avoid biases introduced by a sample overlap, winner’s curse, and weak instruments[29]. Additionally, the mediation proportion of the mediator was assessed using the product of coefficients method. All tests were two-sided and performed using the TwoSampleMR (version 0.5.7), MVMR (version 0.4), MRlap (version 0.0.3.0), and Mendelian Randomization (version 0.9.0) packages in the R software (version 4.0.2). A Bonferroni-corrected P-value less than 0.05/5 (0.01) was identified as statistically significant, and a P-value less than 0.05 was identified as nominally significant. |
| 9 | **Software and pre-registration** |  |  |  |
|  | a) | Name statistical software and package(s), including version and settings used | - | All tests were two-sided and performed using the TwoSampleMR (version 0.5.7), MVMR (version 0.4) and MendelianRandomization (version 0.9.0) packages in the R software (version 4.0.2). |
|  | b) | State whether the study protocol and details were pre-registered (as well as when and where) | - | This is a secondary analysis based on summary statistics from existing, published studies. The ethical approval and informed consent have been obtained by all original studies |
|  | **RESULTS** |  |  |  |
| 10 | **Descriptive data** |  |  |  |
|  | a) | Report the numbers of individuals at each stage of included studies and reasons for exclusion. Consider use of a flow diagram | - | Table 1 |
|  | b) | Report summary statistics for phenotypic exposure(s), outcome(s), and other relevant variables (e.g. means, SDs, proportions) | - | Table 1 |
|  | c) | If the data sources include meta-analyses of previous studies, provide the assessments of heterogeneity across these studies | - | Table 1 |
|  | d) | For two-sample MR:  i.  Provide justification of the similarity of the genetic variant-exposure associations between the exposure and outcome samples  ii.  Provide information on the number of individuals who overlap between the exposure and outcome studies | - | i. we limited our study to European ancestry to avoid bias in population structure.  ii. Overlapping individuals have negligible effects on the power when IVs are strong enough；In addition, an MRlap analysis was performed using the same IVs selection criteria, aiming to avoid biases introduced by a sample overlap, winner’s curse, and weak instruments[29]. |
| 11 | **Main results** |  |  |  |
|  | a) | Report the associations between genetic variant and exposure, and between genetic variant and outcome, preferably on an interpretable scale | - | Supplementary Tables |
|  | b) | Report MR estimates of the relationship between exposure and outcome, and the measures of uncertainty from the MR analysis, on an interpretable scale, such as odds ratio or relative risk per SD difference | - | Most of the resulting space is a report on the contents of the article. |
|  | c) | If relevant, consider translating estimates of relative risk into absolute risk for a meaningful time period | - | - |
|  | d) | Consider plots to visualize results (e.g. forest plot, scatterplot of associations between genetic variants and outcome versus between genetic variants and exposure) | - | Main results are presented in figures. |
| 12 | **Assessment of assumptions** |  |  |  |
|  | a) | Report the assessment of the validity of the assumptions | - | In UVMR and MVMR, Cochran's Q statistic and MR-Egger intercept were performed to evaluate heterogeneity and pleiotropy, respectively. The MR-PRESSO was used to remove the outliers for horizontal pleiotropy and evaluate whether the exclusion of the outlying SNPs influences the causal estimates[27]. In addition, an MRlap analysis was performed using the same IVs selection criteria, aiming to avoid biases introduced by a sample overlap, winner’s curse, and weak instruments[29]. |
|  | b) | Report any additional statistics (e.g., assessments of heterogeneity across genetic variants, such as *I^2^*, Q statistic or E-value) | - | See above |
| 13 | **Sensitivity analyses and additional analyses** |  |  |  |
|  | a) | Report any sensitivity analyses to assess the robustness of the main results to violations of the assumptions | - | See above |
|  | b) | Report results from other sensitivity analyses or additional analyses | - | See above |
|  | c) | Report any assessment of direction of causal relationship (e.g., bidirectional MR) | - | - |
|  | d) | When relevant, report and compare with estimates from non-MR analyses | - | We have described these contents in Discussion. |
|  | e) | Consider additional plots to visualize results (e.g., leave-one-out analyses) | - | - |
|  | **DISCUSSION** |  |  |  |
| 14 | **Key results** | Summarize key results with reference to study objectives | - | Our study systemically evaluated the causal associations between viral hepatitis and psychiatric disorders. We revealed the causal effect of schizophrenia on high CHC risk, whereas no causal association was observed between CHB and psychiatric disorders. A further mediation analysis found that coffee consumption and underweight were the mediators in the effect of schizophrenia on CHC risk. |
| 15 | **Limitations** | Discuss limitations of the study, taking into account the validity of the IV assumptions, other sources of potential bias, and imprecision. Discuss both direction and magnitude of any potential bias and any efforts to address them | - | This study has a few limitations, which should be considered when interpreting our results. First, the selection for partial exposures was conducted on a relax threshold (<5×10-6). However, it was reassuring that the IVs’ F-statistics exceeded 10, indicating that the weak instrument bias should be minimal. Second, partial nominal results in our study were limited to the MR-Egger and weighted median methods rather than the IVW method. Thus, our results should be interpreted cautiously, and further study is required to validate our findings. Third, the study population of our study were limited to European ancestry, indicating that our results should be cautiously interpreted across non-European populations. |
| 16 | **Interpretation** |  |  |  |
|  | a) | Meaning: Give a cautious overall interpretation of results in the context of their limitations and in comparison with other studies | - | The findings of this study revealed that patients with schizophrenia faced a high risk of CHC, and insufficient coffee consumption and underweight could mediate the causal effect of schizophrenia on CHC. The prevention of hepatitis C might be a beneficial strategy for patients with schizophrenia. The right amount of nutrition supplement and coffee consumption might be beneficial lifestyles in preventing high CHC risk in patients with schizophrenia. |
|  | b) | Mechanism: Discuss underlying biological mechanisms that could drive a potential causal relationship between the investigated exposure and the outcome, and whether the gene-environment equivalence assumption is reasonable. Use causal language carefully, clarifying that IV estimates may provide causal effects only under certain assumptions | - | We have described these contents in Discussion. |
|  | c) | Clinical relevance: Discuss whether the results have clinical or public policy relevance, and to what extent they inform effect sizes of possible interventions | - | The findings of this study revealed that patients with schizophrenia faced a high risk of CHC, and insufficient coffee consumption and underweight could mediate the causal effect of schizophrenia on CHC. The prevention of hepatitis C might be a beneficial strategy for patients with schizophrenia. The right amount of nutrition supplement and coffee consumption might be beneficial lifestyles in preventing high CHC risk in patients with schizophrenia. |
| 17 | **Generalizability** | Discuss the generalizability of the study results (a) to other populations, (b) across other exposure periods/timings, and (c) across other levels of exposure |  | - |
|  | **OTHER INFORMATION** |  |  |  |
| 18 | **Funding** | Describe sources of funding and the role of funders in the present study and, if applicable, sources of funding for the databases and original study or studies on which the present study is based | - | We have described these contents in Funding. |
| 19 | **Data and data sharing** | Provide the data used to perform all analyses or report where and how the data can be accessed, and reference these sources in the article. Provide the statistical code needed to reproduce the results in the article, or report whether the code is publicly accessible and if so, where | - | We have described these contents in Availability of data and materials. |
| 20 | **Conflicts of Interest** | All authors should declare all potential conflicts of interest | - | The authors declare that they have no competing interests. |

This checklist is copyrighted by the Equator Network under the Creative Commons Attribution 3.0 Unported (CC BY 3.0) license.

1. Skrivankova VW, Richmond RC, Woolf BAR, Yarmolinsky J, Davies NM, Swanson SA, et al. Strengthening the Reporting of Observational Studies in Epidemiology using Mendelian Randomization (STROBE-MR) Statement. JAMA. 2021;under review.

2. Skrivankova VW, Richmond RC, Woolf BAR, Davies NM, Swanson SA, VanderWeele TJ, et al. Strengthening the Reporting of Observational Studies in Epidemiology using Mendelian Randomisation (STROBE-MR): Explanation and Elaboration. BMJ. 2021;375:n2233.
